# Inhibitory Effects of Adlay Extract on Melanin Production and Cellular Oxygen Stress in B16F10 Melanoma Cells

**DOI:** 10.3390/ijms150916665

**Published:** 2014-09-19

**Authors:** Huey-Chun Huang, Wan-Yu Hsieh, Yu-Lin Niu, Tsong-Min Chang

**Affiliations:** 1 Department of Medical Laboratory Science and Biotechnology, China Medical University, No. 91 Hsueh-Shih Road, Taichung 40402, Taiwan; E-Mail: lchuang@mail.cmu.edu.tw; 2 Department of Applied Cosmetology & Master Program of Cosmetic Sciences, Hungkuang University, No. 1018, Sec. 6, Taiwan Boulevard, Shalu Dist., Taichung City 43302, Taiwan; E-Mail: opency.tw@gmail.com; 3 Niuer International Skincare Science Research Institute, 7F, No. 618, Ruiguang Rd., Neihu Dist., Taipei 114, Taiwan; E-Mail: niuer80@gmail.com

**Keywords:** adlay, MITF, tyrosinase, melanin, ROS

## Abstract

The aim of this study was to determine the effects of adlay extract on melanin production and the antioxidant characteristics of the extract. The seeds were extracted by the supercritical fluid CO_2_ extraction (SFE) method. The effect of adlay extract on melanin production was evaluated using mushroom tyrosinase activity assay, intracellular tyrosinase activity, antioxidant properties and melanin content. Those assays were performed spectrophotometrically. In addition, the expression of melanogenesis-related proteins was determined by western blotting. The results revealed that the adlay extract suppressed intracellular tyrosinase activity and decreased the amount of melanin in B16F10 cells. The adlay extract decreased the expression of microphthalmia-associated transcription factor (MITF), tyrosinase, tyrosinase related protein-1 (TRP-1) and tyrosinase related protein-2 (TRP-2). The extract also exhibited antioxidant characteristics such as free radical scavenging capacity and reducing power. It effectively decreased intracellular reactive oxygen species (ROS) levels in B16F10 cells. We concluded that the adlay extract inhibits melanin production by down-regulation of MITF, tyrosinase, TRP-1 and TRP-2. The antioxidant properties of the extract may also contribute to the inhibition of melanogenesis. The adlay extract can therefore be applied as an inhibitor of melanogenesis and could also act as a natural antioxidant in skin care products.

## 1. Introduction

Melanin is a pigment produced by epidermal melanocytes and is responsible for skin color and for protecting skin from environmental UV damage. In the melanin biosynthesis pathway, tyrosinase catalyzes the rate-limiting steps in which l-tyrosine is hydroxylated to l-3,4-dihydroxyphenylalanine (l-DOPA), and l-DOPA is further oxidized into the corresponding *o*-quinone. Thus, tyrosinase is a major target in screening inhibitors for melanin synthesis. Several skin depigmenting chemicals such as kojic acid [1] and arbutin [2], which act as tyrosinase inhibitors, have been applied in skin whitening products for the treatment or prevention of abnormal skin pigmentation [3]. It has been reported that microphthalmia-associated transcription factor (MITF), tyrosinase related protein-1 (TRP-1) and tyrosinase related protein-2 (TRP-2) also contribute to the production of melanin [4,5]. Additionally, it has been reported that melanogenesis produces hydrogen peroxide (H_2_O_2_) and other reactive oxygen species (ROS) that expose the human melanocytes to high levels of oxidative stress [6]. ROS have been shown to play a significant role in the regulation of melanin synthesis, whereas ROS scavengers and inhibitors of ROS generation may down-regulate UV-induced human melanogenesis [7]. The contribution of ROS to melanogenesis has been studied using antioxidants such as *N*-acetyl cysteine to abolish UVB-induced α-melanocyte stimulating hormone [8]. Stimulation of an endogenous antioxidant, metallothionein, also suppresses melanogenesis in melanocytes [9]. Furthermore, it is reported that UV light radiation causes the synthesis of ROS in skin [10]. Therefore, the use of antioxidants to protect human skin from the harmful effects of UV radiation is a new trend that has attracted increasing interest in the fields of dermatology and skin care products in recent years [11]. The B16F10 murine melanoma cell line was reported to be a good model for studying human melanoma [12]. Hence, we chose this cell line in the present study to evaluate a natural inhibitor of melanogenesis.

Adlay (*Coix lachryma*-*jobi* L. var. *ma*-*yuen* Stapf, Job’s tears) is a traditional Chinese medicinal plant that has been reported to show various pharmacological activities, such as anti-inflammatory [13] and anti-allergic effects [14]. Furthermore, the methanolic extract of adlay seeds inhibits nitric oxide (NO) and O2^−•^ production by activated macrophages [15]. In addition, the adlay seeds’ methanolic extracts exhibited antioxidant activities [16]. However, to date there are no scientific reports on dermatological applications of the seed extract of this plant. The aim of this study is to investigate the inhibitory effect of supercritical fluid CO_2_ extract of adlay seeds on melanogenesis in B16F10 melanoma cells and evaluate the potential antioxidant characteristics of the extract.

## 2. Results and Discussion

### 2.1. Effects of Adlay Extract on Mushroom Tyrosinase Activity and Melanogenesis

**Figure 1:**
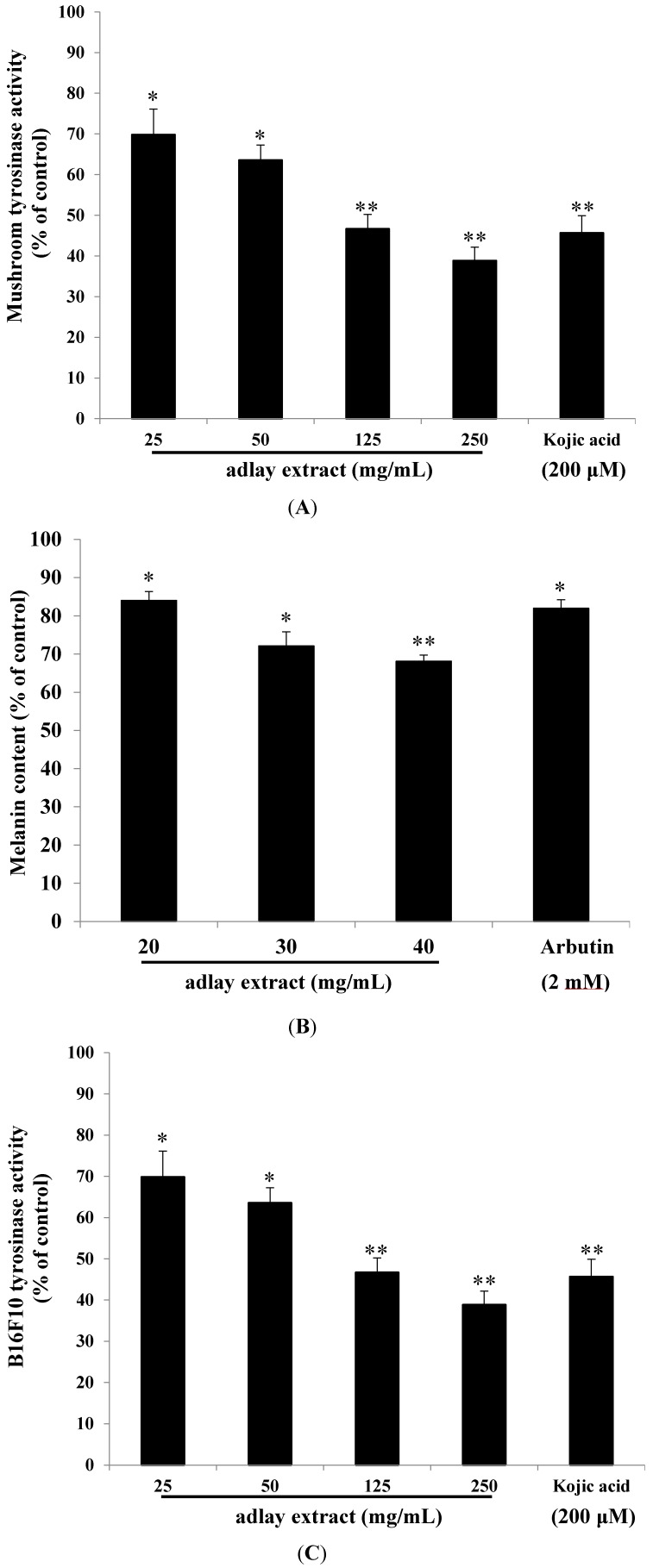
The inhibitory effects of adlay extract on melanogenesis. (**A**) The effects of adlay extract on mushroom tyrosinase activity; (**B**) The effects of adlay extract on melanin content in B16F10 cells; (**C**) The effects of adlay extract on tyrosinase activity in B16F10 cells. The results are presented as percentages of the control, and the data are presented as the mean ± S.D. of three separate experiments. Black bars and lines on each bar indicate the mean and standard deviation. The values are significantly different compared with the control. * *p* < 0.05, ** *p* < 0.01.

To determine the potential inhibitory effect of adlay extract on mushroom tyrosinase activity, enzyme inhibition experiments were carried out in triplicate. Kojic acid was used as a positive standard. The results indicated that mushroom tyrosinase activity was inhibited by various concentrations of adlay extract (25–250 mg/mL). The residual tyrosinase activity was 69.82% ± 6.29%, 63.57% ± 3.66%, 46.68% ± 3.52% and 38.85% ± 3.31% of the control for 25, 50, 125 and 250 mg/mL of adlay extract, respectively. Simultaneously, the tyrosinase activity was inhibited by kojic acid and the remained enzyme activity was 45.67% ± 4.21% of positive control. Mushroom tyrosinase is routinely used for screening potential inhibitors of melanogenesis *in vitro* experiments. Increasing concentration of extract in the reaction mixture exhibited increased inhibitory activity of the enzyme. At 250 mg/mL levels, as much as 60% activity of the enzyme was inhibited. In contrast, kojic acid a well-known inhibitor of mushroom tyrosinase showed an inhibition of about 50% at 200 µM concentrations (0.028 mg/mL) (Figure 1A). Thus, the adlay extract seems to exhibit potent inhibition of mushroom tyrosinase activity.

We then studied the effect of adlay extract on the production of melanin in B16 melanoma cells. Figure 1B shows the results of this study. For comparison, the effect of arbutin, a skin-lightening compound on the melanin production in this cell line at 2 mM concentration (0.545 mg/mL) is given. As is evident, increased concentrations of adlay extract inhibited the melanin production increasingly with an IC_50_ value of 61.21 mg/mL. Thus adlay extract seems to be efficient in inhibiting melanin production in the B16 melanoma cell line.

In order to see if the above inhibition is caused by the inhibition of endogenous tyrosinase activity, intracellular tyrosinase activity was determined after treating the cells with various concentrations of the adlay extract. Again, increasing concentrations of adlay extract caused increased inhibition of intracellular tyrosinase activity with an IC_50_ value of 36.31 mg/mL (Figure 1C). Thus, the results presented in Figure 1 clearly indicate that adlay extracts not only possess potent inhibition of tyrosinase activity, but also have significant ability to reduce the melanin production in cultured cells.

One might argue that the reduced melanin production and reduced intracellular tyrosinase activity observed with B16 melanoma cells could be due to the cytotoxicity of the adlay extracts, which might have killed the cells and reduced the viable cells there by showing reduced melanin production and reduced enzyme activity. To assess this possibility, the viability of the adlay extract treated cells were determined by MTT assay. MTT is a pale yellow compound that is converted by living cells to a dark blue formazan product, in contrast, dead cells do not perform this reaction and hence remain yellow colored. Therefore, living cells will appear blue while dead cells appear yellow after treatment with MTT. Experiments conducted with MTT treatment on B16 melanoma cells with various concentrations of the SFE (20, 30 and 40 mg/mL) for 24 h did not show any significant difference in the viability of the cells thereby indicating that the adlay extract is not cytotoxic to B16F10 cells (data not shown). In the experiments described here, alpha-melanocyte-stimulating hormone (α-MSH) was used as a cAMP inducer to stimulate melanin synthesis. It is reported that α-MSH can bind melanocortin 1 receptor (MC1R) and activate adenylate cyclase, which in turn catalyzes ATP to cAMP and increases intracellular cAMP levels [17]. The results showed that the adlay extract inhibited melanogenesis induced by α-MSH-mediated intracellular cAMP up-regulation. Furthermore, some chemical depigmenting agents show side effects such as the genotoxic effect of arbutin [18] and pigmented contact dermatitis due to kojic acid [19]. Hence, searching for a safe and effective skin whitening agent remains a goal in the fields of cosmetic research and development.

To further confirm these findings and to assess the effect of adlay extracts on the activity of other melanogenic enzymes, we conducted the following studies. It is now well established that apart from tyrosinase, a number of other proteins also participate in melanogenesis. TRP-1, which converts dopachrome to 5,6-dihydroxyindole-2-carboxylic acid and TRP-2, which oxidizes 5,6-dihydroxyindole-2-carboxylic acid, are two other proteins that are associated with melanogenesis. MITF is another protein that plays a crucial role in melanogenesis by being a major transcriptional regulator of tyrosinase, TRP-1 and TRP-2 genes in animals. Therefore, we tested the levels of these proteins by western blot analysis after expose to adlay extracts.

**Figure 2:**
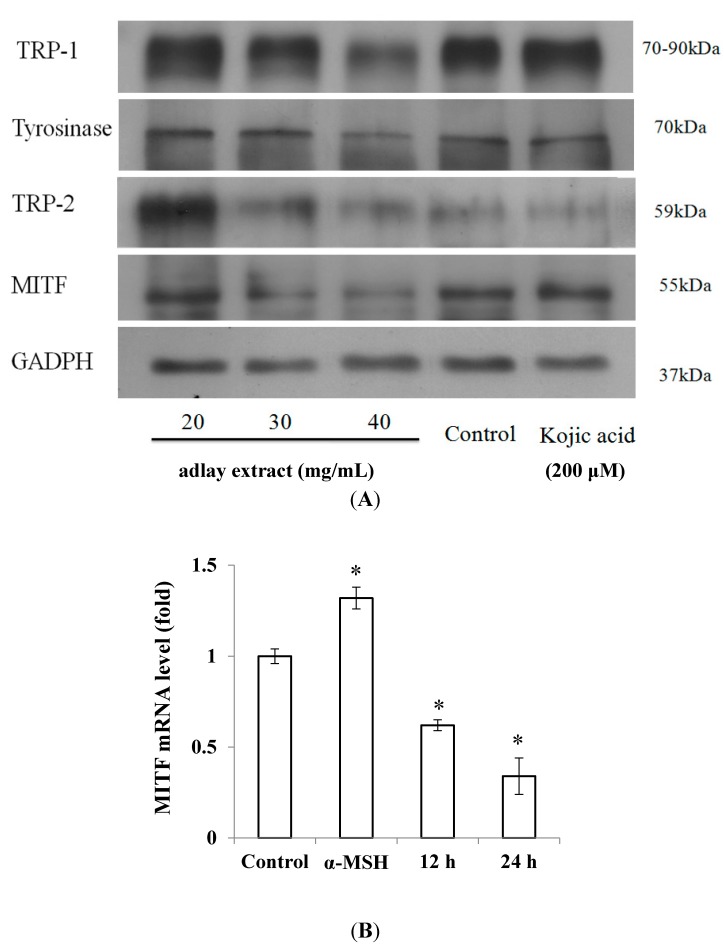
The effects of adlay extract on melanogenesis-related protein expression levels. (**A**) Western blotting of cellular proteins in B16F10 cells; (**B**) Total RNA from B16F10 cells treated with adlay extract (40 mg/mL) collected at the indicated time. microphthalmia-associated transcription factor (MITF) mRNA levels were examined by real time RT-PCR, using glyceraldehyde 3-phosphate dehydrogenase (GAPDH) as an internal control. (* *p* < 0.05 *vs.* control).

Figure 2A shows the western blot analysis of tyrosinase, TRP-1, TRP-2 and MITF along with a control protein GAPDH after treatment with adlay extracts and kojic acid. As expected, different treatments did not affect the levels of GAPDH. The kojic acid, a potent inhibitor of tyrosinase also did not affect the expression levels of tyrosinase, TRP-1, TRP-2 and MITF. However, different concentrations of adlay extract affected the expression levels of these proteins.

To assess the effect of adlay extracts on the cellular melanin production, we determined the mRNA levels of MITF in cells treated with α-MSH and adlay extracts. The α-MSH treated cells showed increased levels of MITF as expected. Interestingly adlay extract treated cells showed reduction in the expression of MITF mRNA levels (Figure 2B).

### 2.2. Antioxidant Characteristics of the Adlay Extract

**Figure 3:**
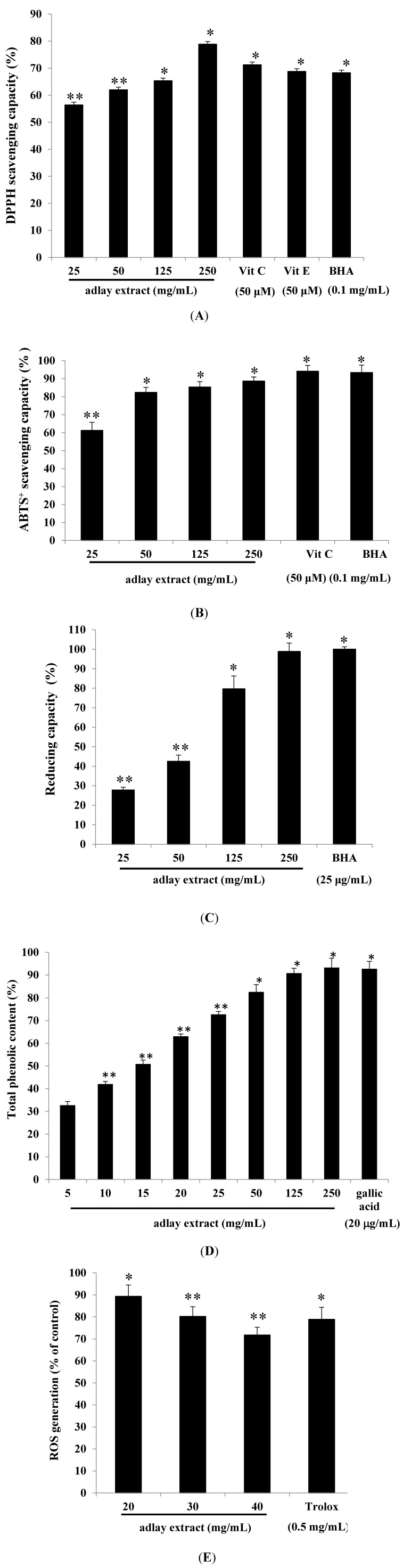
The antioxidant characteristics of adlay extract. (**A**) DPPH scavenging capacity assay; (**B**) ABTS^+^ radical scavenging activity assay; (**C**) Determination of reducing capacity; (**D**) Measurement of total phenolic content; (**E**) ROS level assay. Different concentrations of the adlay extract and positive standards were used in the above assays. The results are expressed as percentages of the control. The data are presented as the mean ± S.D. * *p* < 0.05, ** *p* < 0.01.

We then focused out attention on the antioxidant properties of adlay extracts. To this end, we carried out the radical scavenging assay using 2,2-diphenyl-1-picrylhydrazyl (DPPH). DPPH is a stable free radical that can interact with different antioxidant molecules. During the process, it gets reduced to a colorless compound. Therefore, antioxidant properties of many compounds can be easily determined by their ability to reduce this colored compound by monitoring the decrease in absorbance at 517 nm using a UV-visible spectrophotometer. For control, we used vitamin C, vitamin E and butylated hydroxyanisole (BHA). The results shown in Figure 3A clearly indicate that adlay extracts possess antioxidant properties.

The ABTS^+^ free radical was used to determine the free radical scavenging activity of the adlay extract. Interaction of antioxidants with ABTS^+^ transfer hydrogen atoms to ABTS^+^ thus neutralizing its free radical character. The adlay extract showed similar ABTS^+^ free radical scavenging activities as that of Vitamin C or BHA (Figure 3B). As shown in Figure 3C, adlay extracts at higher concentration exhibited the same reducing capacity as that of BHA (25 µg/mL). The antioxidant activity of phenolic compounds is probably due to their redox properties, which can play an important role in adsorbing and neutralizing free radicals, quenching singlet and triplet oxygen, or decomposing peroxides. At lower concentration range (5–125 mg/mL), increasing concentration of extract shows a proportional increase in phenolic content. It was found that higher concentrations of the extract (125 and 250 mg/mL) show similar phenolic content as that of gallic acid (Figure 3D). This is probably due to some bioactive compounds such as polyphenols including tannins and flavonoid existed in the extracts.

Therefore, we studied the effect of adlay extract on intracellular ROS levels. The study was conducted with the use of DCFH-DA (2',7'-dichlorofluorescein diacetate), which diffuses through the cell membrane easily and is hydrolyzed by the endogenous esterases to DCFH. DCFH thus formed reacts with ROS such as H_2_O_2_ to generate DCF. To confirm the antioxidant capacity of adlay extract in a cellular model, evaluation of intracellular ROS levels was done. Rapid increases in DCF indicate oxidation of DCFH by intracellular radicals (Figure 3E).

When exposed to UV light or environmental oxidizing agents, human skin experiences oxidative stress. It has been reported that ultraviolet irradiation induces the formation of ROS in cutaneous tissue [20], provoking damage such as enzyme inactivation and lipid peroxidation. The search for antioxidants with skin-depigmenting capabilities is driven by the hypothesis that oxidative stress resulting from UV-irradiation may contribute to the stimulation of melanogenesis. In addition, redox agents may also influence melanin production by interacting with the copper ion at the active site of tyrosinase or with *o*-quinones to block the oxidative polymerization of melanin intermediates. Interestingly, plant extracts such as chestnut flower extract [21] and *Paeonia suffruticosa* [22] have been reported to show antioxidant and antimelanogenic properties. Our research indicates that the apparent antioxidant capacity of adlay extract may contribute to its depigmenting activity and could be included in cosmetic formulations of skin care products. Certainly, the efficacy of adlay extract-containing products should be evaluated by applying to human study in the near future.

## 3. Experimental Section

### 3.1. Chemicals and Reagents

All chemical reagents were purchased from Sigma Chemical Co. (St. Louis, MO, USA). The antibodies were obtained from Santa Cruz Biotech (Santa Cruz, CA, USA), and the ECL reagent was from Millipore (Milford, MA, USA).

### 3.2. Supercritical Fluid CO_2_ Extraction (SFE) of Adlay Seeds

The adlay seeds were harvested in May 2012 from Nantou County, Taiwan. The seeds were washed, exposed to sunlight and air-dried for one day. The seeds were then dried at 80 °C for 2 h in an oven. The dried seeds were pulverized to a fine powder with a committed mill (Retsch Ultra Centrifugal Mill and Sieving Machine, Type ZM1, Haan, Germany). The adlay seed powder (75 g) was placed in the extraction vessel (200 mL) of the supercritical fluid CO_2_ extraction apparatus (SFE-400S-2000, Metal Industries Research and Development Centre; MIRDC; Kaohsiung, Taiwan). The extraction was performed with 10% co-solvent of ethanol in supercritical fluid CO_2_ (flow rate, 5.0 mL/min) at 5000 psi (350 bar) at a temperature of 50 °C for 2 h. The extracts were dried on a rotary evaporator at 40 °C under reduced pressure. The concentrated SFEs were weighed and stored at −20 °C. In the following experiments, the SFEs were re-dissolved in dimethyl sulfoxide (DMSO), as indicated, and the final concentrations of DMSO in the cellular experiments were less than 1%.

### 3.3. Inhibitory Effects of Adlay Extract on Melanogenesis

Inhibition assays of mushroom tyrosinase activities were conducted as previously described [23]. A reaction mixture containing 200 units (in 10 μL) of mushroom tyrosinase and 5 mM DOPA in 50 mM sodium phosphate buffer pH 6.8 was incubated in 96 well microtiter plate at 37 °C for 30 min. The reaction was started by the addition of enzyme. Following incubation, the amount of dopachrome produced in the reaction mixture was determined spectrophotometrically at 490 nm (OD_490_) using a microplate reader. The inhibition percentage at three doses for each experiment was calculated by the following equation: inhibition percentage of tyrosinase activity (%) = (B − A) ÷ A × 100, where B is the mean of the measured OD_490_ values of the blank control, and A is the mean of the measured OD_490_ values for the adlay extract treated group.

The B16F10 cells (ATCC CRL-6475, BCRC60031) were obtained from the Bioresource Collection and Research Center (BCRC), Taiwan. The cells were maintained in DMEM (Hyclone, Logan, UT, USA) supplemented with 10% fetal bovine serum and 1% antibiotics at 37 °C, 5% CO_2_ in a humidified incubator. The melanin content was measured as previously described by Tsuboi *et al.* [24], while the cellular tyrosinase activity was determined as described in an earlier publication with some modifications [25]. The cells were pretreated with α-MSH (100 nM) for 24 h and treated with either the adlay extract or arbutin for a further 24 h, then the melanin content or intracellular tyrosinase activity was measured. After treatment, the cells were detached by incubation in trypsin/ethylenediaminetetraacetic acid (EDTA). After precipitation at 900 r.p.m for 5 min, cell pellets containing a known number of cells were solubilized in 1 N NaOH at 60 °C for 60 min. The melanin content was determined by spectrophotometric analysis by measuring its absorbance of 405 nm. The intracellular tyrosinase activity was determined as follows: Cell extracts (100 μL) were mixed with freshly prepared l-DOPA solution (0.1% in phosphate-buffered saline) and incubated at 37 °C. The absorbance of this solution at 490 nm was measured with microplate reader Gen 5™ (BIO-TEK Instrument, Vermont, VT, USA) to estimate the production of dopachrome. Corrections were made for auto-oxidation of l-DOPA.

The western blotting experiments were performed as outlined in our earlier study [26]. The relative amounts of MITF, tyrosinase, TRP-1 and TRP-2 compared with total glyceraldehyde 3-phosphate dehydrogenase (GAPDH) were calculated and analyzed with Multi Gauge 3.0 software (Fuji, Tokyo, Japan).

### 3.4. RT-PCR

RNA samples were reverse-transcribed for 120 min at 37 °C with High Capacity cDNA Reverse Transcription Kit according to the standard protocol of the supplier (Applied Biosystems, Foster City, CA, USA). Quantitative PCR was performed by as follows: 10 min at 95 °C, 40 cycles of 15 s at 95 °C, 1 min at 60 °C using 2× Power SYBR Green PCR Master Mix (Applied Biosystems, Foster City, CA, USA) and 200 nM of forward and reverse primers (MITF gene forward primer: GGCCAAGGCAGAGCAACTT; MITF gene reverse primer: GCCCATGGTGGCAAGCT; GAPDH gene forward primer: ATCCCATCACCATCTTCCAG; GAPDH gene reverse primer: CCATCACGCCACAGTTTCC). Each assay was carried out on an Applied Biosystems 7300 Real-Time PCR system in triplicate and expression fold-changes were derived using the comparative C_t_ method. Relative quantification was calculated using GAPDH as an endogenous control. Fold changes were calculated as the following Equations (1) and (2):

Fold change = 2^−∆∆Ct^ (1)

2^−∆∆Ct^ = [(C_t_ gene of interest − C_t_ internal control) treated sample − (C_t_ gene of interest − C_t_ internal control) untreated control)] (2)

### 3.5. Antioxidant Characteristics of Adlay Extract

The antioxidant characteristics of adlay seed SFE was first determined by measuring the DPPH scavenging ability as previously described [27]. The ABTS decolorization assays were conducted as previously described. The ABTS^+^ chromophore necessary for the assay was generated from a 7 mM stock solution of ABTS by mixing with 2.45 mM potassium persulfate solution and leaving the mixture in the dark for 6 h before use. One mL of the ABTS^+^ thus formed was incubated with different concentrations of adlay extract for 10 min and the absorbance change was quantified at 734 nm. The ABTS^+^ scavenging capacity of adlay extract thus determined was compared with that of vitamin C (50 μM) and butylated hydroxyanisole (BHA; 0.1 mg/mL) [28].

The reducing capacity was determined according to previously published methods [29]. The test sample was mixed with phosphate buffer (2.5 mL, 0.2 M, pH 6.6) and potassium ferricyanide [K_3_Fe(CN)_6_] (2.5 mL, 1% *w*/*v*). The mixture was incubated at 50 °C for 20 min. At the end of the reaction, 2.5 mL of trichloroacetic acid (10% *w*/*v*) was added to the mixture, and it was centrifuged at 1000× *g* for 10 min. The supernatant (2.5 mL) was treated with distilled water (2.5 mL) and FeCl_3_ (0.5 mL, 0.1% *w*/*v*), and the absorbance of the final solution was measured at 700 nm in a UV-Vis spectrophotometer. The content of total phenolics was determined with the Folin-Ciocalteu reagent [30] and gallic acid (20 μg/mL) was used as a positive standard. Different concentrations of samples were prepared in 80% of methanol. One-hundred microliters of sample were dissolved in 500 μL (1/10 dilution) of the Folin-Ciocalteu reagent and 1 mL of distilled water. The solutions were mixed and incubated at room temperature. After 1 min, 1.5 mL of 20% sodium carbonate solution was added. The final mixture was shaken and then incubated for 2 h in the dark at room temperature. The absorbance of samples was measured at 760 nm.

The cellular ROS levels were determined with the 2',7'-dichlorofluorescein diacetate (DCFH-DA) method [31]. B16F10 melanoma cells were cultured in 24-well plates (5 × 10^4^ cells in 1 mL of DMEM medium) and treated with various concentrations of adlay extract for 24 h. The cells were then incubated with 24 mM H_2_O_2_ at 37 °C for 30 min. After incubation, DCFH-DA was added to the wells, and the cells were cultured for 30 min. Following this treatment, the cells were washed with phosphate-buffered saline and trypsinized with trypsin/EDTA, and the fluorescence intensities of DCF were measured at excitation wavelength 504 nm and emission wavelength 524 nm using a fluorescent reader Fluoroskan Ascent (Thermo Scientific, Vantaa, Finland). The data were analyzed with Ascent software (Thermo Scientific, Vantaa, Finland).

### 3.6. Statistical Analysis

Statistical analysis of the experimental data points was performed by the ANOVA test, which was used for comparison of measured data using SPSS 12.0 statistical software (SPSS INC. Chicago, IL, USA). Differences were considered to be statistically significant at *p* < 0.05.

## 4. Conclusions

The current study constitutes the first report on the potential inhibitory effect of adlay extract on the melanin biosynthesis in B16 melanoma cells. Our studies also demonstrate the presence of powerful antioxidant activities in this extract. The inhibition of melanin production observed in the present study could be due a number of factors such as inhibition of tyrosinase, TRP-1, and TRP-2, the reduced expression of MITF, and due to depletion of cellular ROS. Based on our results, we forecast that adlay extract could be used as powerful skin lightening compound possessing antioxidant properties in the skin care industry.
